# Citrus Consumption and Risk of Melanoma: A Dose-Response Meta-Analysis of Prospective Cohort Studies

**DOI:** 10.3389/fnut.2022.904957

**Published:** 2022-06-20

**Authors:** Xuexian Fang, Dan Han, Jun Yang, Fulun Li, Xinbing Sui

**Affiliations:** ^1^Department of Nutrition and Toxicology, School of Public Health, Hangzhou Normal University, Hangzhou, China; ^2^Department of Nutrition and Food Safety, Zhejiang Provincial Center for Disease Control and Prevention, Hangzhou, China; ^3^Department of Dermatology, Yueyang Hospital of Integrated Traditional Chinese and Western Medicine, Shanghai University of Traditional Chinese Medicine, Shanghai, China; ^4^School of Pharmacy and Department of Medical Oncology, The Affiliated Hospital of Hangzhou Normal University, Hangzhou Normal University, Hangzhou, China

**Keywords:** citrus, melanoma, skin cancer, meta-analysis, dose-response

## Abstract

**Background:**

Epidemiological studies of citrus consumption in relation to melanoma risk have yielded conflicting results. This meta-analysis was performed to investigate the dose-response association between citrus consumption and risk of melanoma.

**Methods:**

Relevant prospective cohort studies were identified by searching PubMed, Embase, Scopus, and Web of Science databases up to February 28th, 2022. Results from individual studies were pooled using a random-effects model.

**Results:**

Five prospective studies, with 8,836 melanoma cases and 977,558 participants, were included in the meta-analysis. A significantly increased risk of melanoma was associated with the highest categories of either total citrus products (RR: 1.20; 95% CI: 1.01–1.42) or citrus fruit consumption (1.15; 1.04–1.28), but consumption of citrus juice was not associated with melanoma risk (1.08; 0.97–1.21). The dose-response analyses revealed that for per 1 serving/day increase in total citrus or citrus fruit consumption, the risk of melanoma increased by 9 and 12%, respectively. An inverted U-shaped curvilinear relationship, but not linear association, was observed between citrus juice consumption and melanoma risk.

**Conclusions:**

Citrus consumption was generally associated with a greater risk of malignant melanoma. Our findings may have important public health implications with respect to preventing melanoma.

## Introduction

The global incidence of malignant melanoma has increased steadily over the past several decades (1). Currently, melanoma is regarded as the fifth most common cancer in the United States, accounting for 6 and 5% of the total cancer cases in males and females, respectively (2). Melanoma is also a potentially lethal malignancy, causing a large majority of skin cancer deaths. Given such significant incidence and mortality of melanoma, there is an urgent need to develop primary prevention strategies.

High exposure to solar ultraviolet (UV) is thought to play a fundamentally important role in the development of malignant melanoma (3). Such photocarcinogenesis is induced through DNA damage, oxidative stress, and immunosuppression (4). Psoralens, a group of naturally occurring furocoumarins, have potential photocarcinogenic properties and are abundantly found in citrus products (5). Thus, several large population-based studies have examined the association between citrus consumption and risk of melanoma since 2015. However, the overall conclusion remains controversial, as the results of these studies yielded inconsistent.

Understanding the exact influence of high citrus consumption on melanoma risk have important public health implications with respect to establishing clear dietary guidelines to prevent melanoma. Therefore, we conducted the current meta-analysis of all available prospective cohort studies to quantify the dose-response relationships between dietary consumption of citrus products, including total citrus, citrus fruits, and citrus juice, and melanoma risk.

## Methods

This meta-analysis was designed, implemented, analyzed, and reported in accordance with the Meta-analysis of Observational Studies in Epidemiology (MOOSE) protocol (6).

### Literature Search and Selection

We systematically searched papers that published until February 28th, 2022 in the databases PubMed, Embase, Scopus, and Web of Science. The following keywords were used in the systematic search: (citrus OR “citrus aurantifolia” OR “citrus sinensis” OR “citrus paradisi” OR “citrus juice” OR lemon OR grapefruit OR orange) AND (melanoma OR “melanotic freckle” OR “skin cancer”). Our search was restricted to observational studies conducted in humans, and no restriction was applied on language of the publications. Additional articles were also identified by reviewing the references cited within the retrieved relevant studies. The literature search was conducted by two investigators independently.

Studies were considered eligible if they satisfied the following four criteria: (1) prospective study design of adults (aged > 18 y); (2) the exposure of interest was dietary intake of citrus, including citrus fruit and citrus juice; (3) the outcome was melanoma; and (4) the authors reported risk estimates with 95% confidence intervals (95% CI). Retrospective studies, animal studies, commentaries, reviews, and meta-analyses were excluded, as well as studies that focused on other skin malignancies. To ensure the correct identification of eligible studies, a two-step selection process was performed. Two investigators conducted an initial screening of all titles and/or abstracts independently; the full text of each potentially relevant paper was then evaluated. Any discrepancies were solved by consensus or by a third senior reviewer.

### Data Extraction

Data were extracted using a standardized data collection form. We extracted the following information from every included study: first author and year of publication, the study's location, the study's name (where applicable), the duration of follow-up, gender distribution, sample size (the number of cases and participants), the method used to assess dietary intake (food-frequency questionnaire, 24-h recall, or other), the dietary categories of citrus consumption, and the corresponding risk estimates with 95% CI. We extracted the risk estimates from only the fully adjusted models.

The quality of these included studies was evaluated according to the Newcastle-Ottawa scale for non-randomized studies (7). This scale assigns a maximum of 9 points to each study as follows: 4 for the selection of participants and measurement of exposure, 2 for comparability, and 3 for assessment of outcomes and adequate follow-up. A score of 0–3, 4–6, or 7–9 was regarded as low, moderate, or high quality, respectively.

### Statistical Analysis

Relative risks (RRs) or odds ratios (ORs) with 95% CIs were used as the common measure of association across studies. We calculated the summarized RRs and their corresponding 95% CIs using a random-effects model, which incorporates both within- and between-study variability (8). When studies reported data separately by citrus type, we pooled the relative risks using a fixed-effects model before inclusion in the meta-analysis.

Due to the relatively wide range of definitions for the exposure categories in the included articles, we performed a dose-response analysis based on a 1 serving/day increase in consumption of total citrus, citrus fruit, and citrus juice, in addition to the comparison between the highest and lowest categories. When the consumption was expressed in grams, we converted the amount by considering the average weight: 100 g for a serving of total citrus or citrus fruit, and 50 g for a serving of citrus juice. The method of dose-response meta-analysis was proposed by Greenland and Longnecker and the publicly available code written by Greenland and Longnecker (9) and Orsini et al. (10). The categories of citrus consumption, distributions of cases and person-years, and RR and 95% CI were extracted. If neither median nor mean values were reported, we used the categorical midpoint. If the highest or lowest category was open-ended, the midpoint of the category was estimated by assuming that the width of the category was the same as the next adjacent category. In addition, we evaluated the potential non-linear associations between dietary intake of total citrus, citrus fruit, and citrus juice and melanoma risk using restricted cubic splines, with three knots at the 10th, 50th, and 90th percentiles of the distribution (11).

Heterogeneity among the studies was evaluated using the *I*^2^ statistic, with values of 25, 50, and 75% representing low, moderate, and high degrees of heterogeneity, respectively (12, 13). We assessed publication bias with Begg's rank correlation test and Egger's linear regression test (14–16). All statistical analyses were performed using STATA version 14.0 software (StataCorp, TX). Except where noted otherwise, a *P*-value < 0.05 was considered significant.

## Results

### Study Selection and Characteristics

The flow diagram for the study selection process can be seen in Supplementary Figure 1. Using the search strategy, we identified 528 articles from PubMed, 1,315 articles from Embase, 920 articles from Scopus, and 769 articles from Web of Science. After removing duplicates, 605 articles were checked for eligibility based on the title and abstract. Finally, five articles, published from 2015 through 2021, were included for this meta-analysis, with data from six independent population-based studies (17–21).

Table 1 summarizes the detailed characteristics of eligible studies, all of which had a prospective cohort design. Four studies were conducted in the United States (19–21), and two in Europe (17, 18). In total, 977,558 participants and 8,836 melanoma cases were identified during the follow-up periods. Citrus consumption was assessed by a food frequency questionnaire (FFQ) in all these studies except for one that used a 24-h dietary recall. Assessment of study quality yielded an average score of 8.6 (Supplementary Table 1).

**Table 1:** Characteristics of the included prospective cohort studies.

**Source**	**Location**	**Study Name**	**Sex**	**Age, y**	**Follow-up, y**	**Cases/Participants**	**Dietary assessment**	**Quality**
Mahamat-Saleh et al. (17)	Europe	EPIC	M/F	52.3 ± 9.3	13.7	1,371/270,112	Validated FFQ	9
Marley et al. (18)	UK	UK Biobank	M/F	56.2 ± 7.9	NA	1,592/198,964	24-h recall	8
Melough et al. (19)	US	WHI	F	63.4 ± 7.2	15.7	956/56,205	Validated FFQ	9
Melough et al. (20)	US	NIH-AARP	M/F	62.0	15.5	3,894/388,467	Validated FFQ	9
Wu et al. (21)	US	NHS	F	60.1	20.2	1,023/63,810	Validated FFQ	9
		HPFS	M	60.7	17.1	817/41,622	Validated FFQ	

*AARP, American Association of Retired Persons; EPIC, European Prospective Investigation into Cancer; F, Female; FFQ, Food Frequency Questionnaire; HPFS, Health Professionals Follow-up Study; NHS, Nurses' Health Study; M, Male; NIH, National Institutes of Health; UK, United Kingdom; US, United States*.

### Total Citrus Consumption and Melanoma Risk

**Figure 1:**
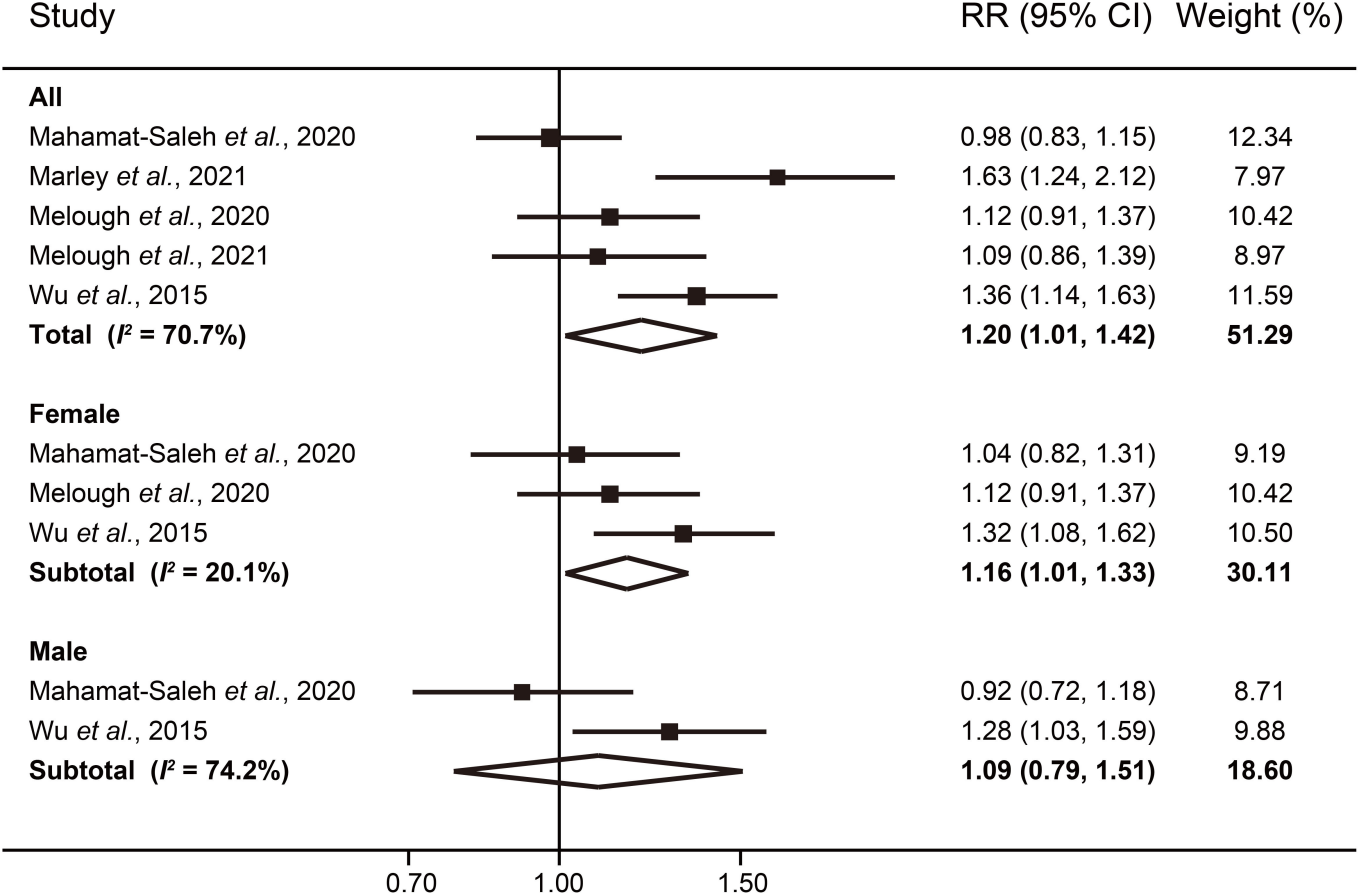
Forest plot of melanoma risk for highest vs. lowest categories of total citrus intake.

The multivariable-adjusted RRs of melanoma risk for the highest vs. the lowest category of total citrus consumption in each study, and for all studies combined, are shown in Figure 1. The pooled RR of PD was 1.20 (95% CI: 1.01–1.42), suggesting higher consumption is significantly associated with an increased risk of developing melanoma. We found evidence of between-study heterogeneity (*I*^2^ = 70.7%) but not publication bias (*P* = 0.46 for Begg's test; *P* = 0.50 for Egger's test). Interestingly, when stratified by gender, the correlation remained statistically significant only among women (RR: 1.16; 95% CI: 1.01–1.33) with attenuated heterogeneity (*I*^2^ = 20.1%).

### Citrus Fruits Consumption and Melanoma Risk

**Figure 2:**
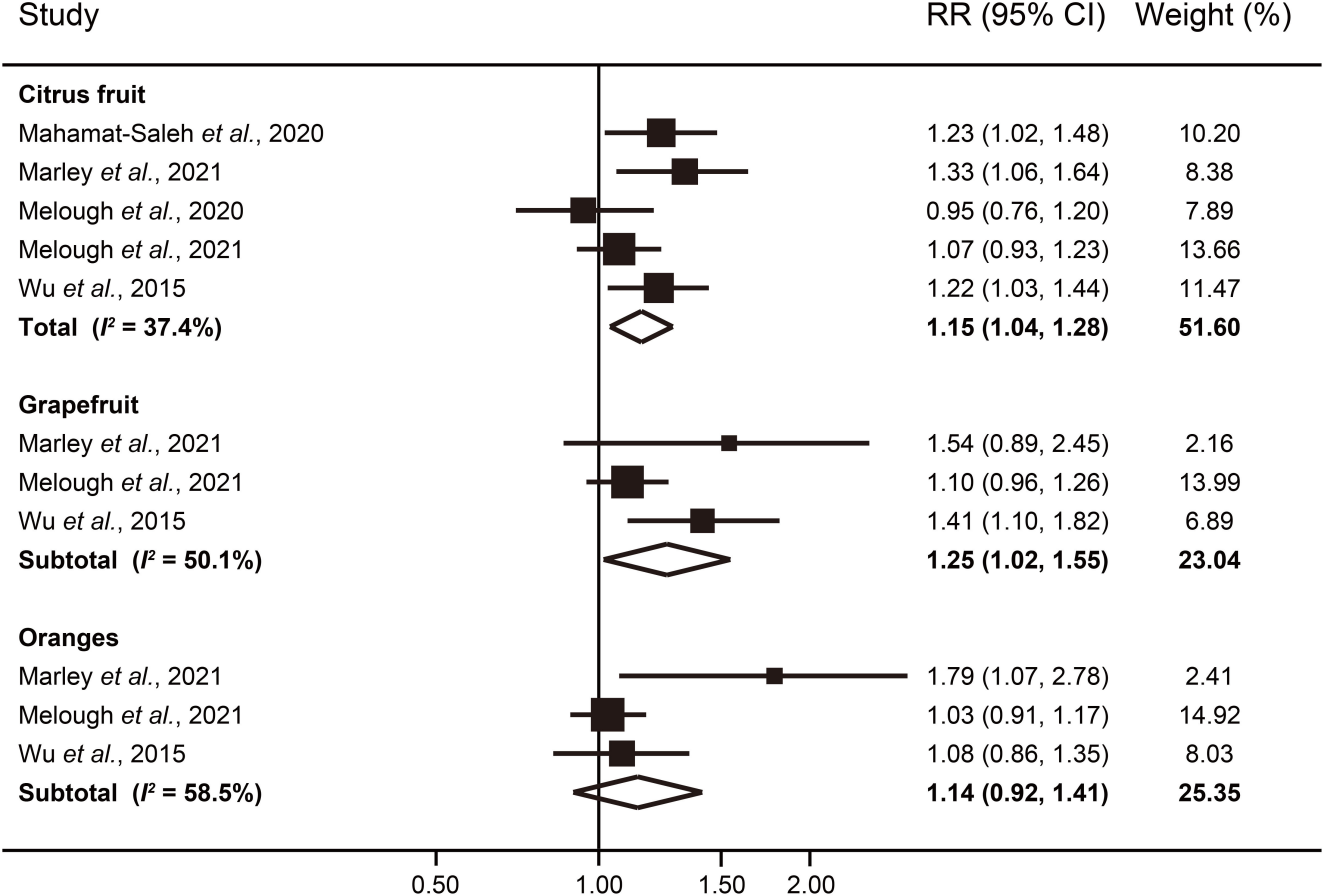
Forest plot of melanoma risk for highest vs. lowest categories of citrus fruit intake.

In the fully adjusted models, participants in the highest category of citrus fruit consumption had a significantly increased risk of melanoma (RR: 1.15; 95% CI: 1.04–1.28), with moderate heterogeneity (*I*^2^ = 37.4%) (Figure 2). Neither the Begg's test nor the Egger's test for publication bias reached significance (*P* = 1.00 and 0.87, respectively). Among individual citrus fruits, grapefruit showed a more apparent association with melanoma risk (RR: 1.25; 95% CI: 1.02–1.55; *I*^2^ = 50.1%). Consumption of oranges was not significantly associated with a higher risk of melanoma (RR: 1.14; 95% CI: 0.92–1.41; *I*^2^ = 58.5%).

### Citrus Juice Consumption and Melanoma Risk

**Figure 3:**
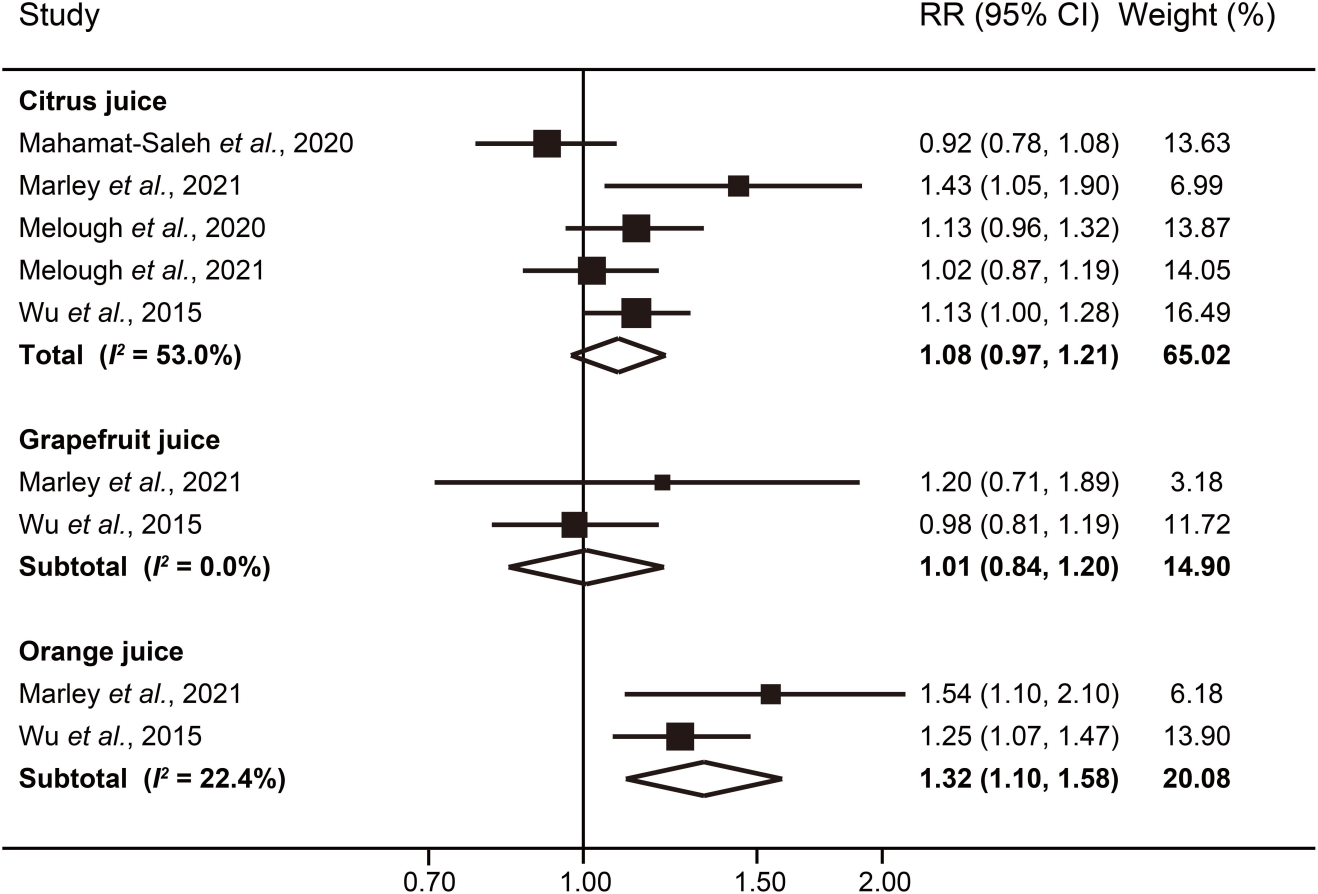
Forest plot of melanoma risk for highest vs. lowest categories of citrus juice intake.

The effect of citrus juice consumption on melanoma risk was also analyzed. However, we did not observe consistent significant association between consumption of citrus juice and melanoma risk (RR: 1.08; 95% CI: 0.97–1.21; *I*^2^ = 53.0%), without evidence of publication bias (*P* = 1.00 for Begg's test; *P* = 0.72 for Egger's test) (Figure 3).Two studies provided results for subtypes of citrus juice (18, 21). For consumption of grapefruit juice, the pooled RR was 1.01 (95% CI: 0.84–1.20; *I*^2^ = 0.0%), comparing the highest category with the lowest. However, significant association was seen between orange juice consumption and melanoma risk; the pooled RR comparing the highest with the lowest levels of orange juice consumption was 1.32 (95% CI: 1.10–1.58; *I*^2^ = 22.4%).

### Dose-Response Analyses

**Figure 4:**
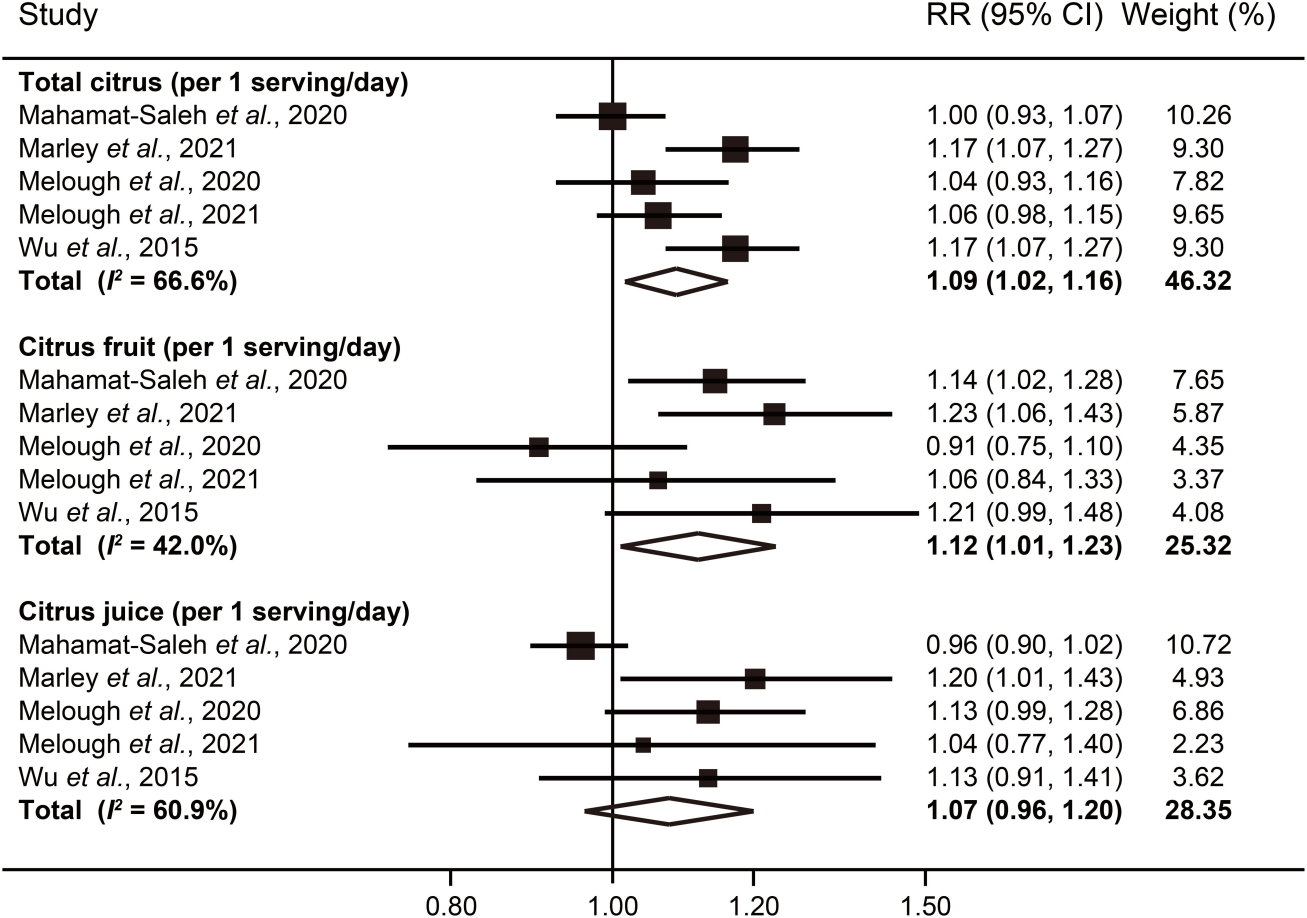
Forest plot of melanoma risk for per 1 serving/day increased intake of total citrus, citrus fruit, and citrus juice.

**Figure 5:**
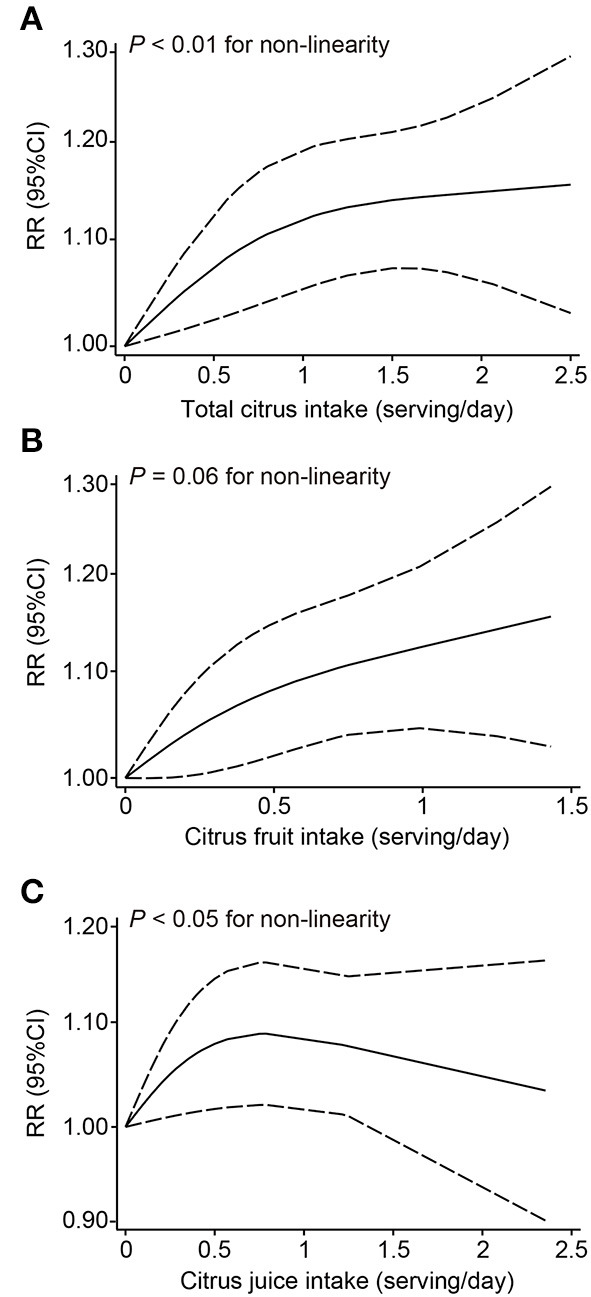
Dose-response analyses of the non-linear association between dietary intake of total citrus **(A)**, citrus fruit **(B)**, and citrus juice **(C)** and the risk of melanoma.

In our dose-response analysis, the results revealed that each 1 serving/day increase in total citrus consumption was associated with a 9% higher risk of melanoma (RR: 1.09; 95% CI: 1.02–1.16; *I*^2^ = 66.6%) (Figure 4). In addition, a significant curvilinear association was also found using the restricted cubic splines model (*P* < 0.01 for non-linearity) (Figure 5). When we examined the melanoma risk associated with a 1 serving/day increase in citrus fruit consumption, the pooled RR was 1.12 (95% CI: 1.01–1.23), with relatively low heterogeneity (*I*^2^ = 42.0%) (Figure 4). However, there was no significant non-linear relationship found between citrus fruit consumption and risk of melanoma (*P* = 0.06 for non-linearity) (Figure 5). The association of citrus juice consumption with melanoma risk was not linear (RR: 1.07; 95% CI: 0.96–1.20; *I*^2^ = 60.9%) (Figure 4), but in an inverted U-shaped manner (*P* < 0.05 for non-linearity), with the highest risk at ~0.5–1 serving per day (Figure 5).

## Discussion

In this meta-analysis of prospective cohort studies, we observed a positive association with increased melanoma risk for higher consumption of citrus, especially of citrus fruits. Significantly higher risks of 9 and 12% were seen for melanoma with every additional 1 serving/day of total citrus and citrus fruits consumed, respectively. Although no significant trend for citrus juice consumption in linear dose-response analysis, an inverted U-shaped curvilinear association was shown. To the best of our knowledge, this is the first meta-analysis providing a comprehensive insight into the quantitative association between consumption of citrus products and melanoma risk.

Citrus products are widely popular foods, but are also rich sources of psoralen, a naturally occurring furocoumarin used to kill foreign pathogens (22). In the past decades, a series of experimental studies have demonstrated that psoralen is photoactive, and can induce gene mutation by intercalating DNA (23, 24). Oral psoralen has served in UV radiation dependent photochemotherapy of psoriasis and other skin diseases since 1970s (25). However, the treatment is also known to be photocarcinogenic (26), and has raised concern for whether consumption of psoralen-rich citrus may increase risk of cutaneous melanoma.

Since 2015, five large-scale prospective cohort studies have investigated the hypothesis, but yielded mixed results. Data from the Nurses' Health Study (NHS) and Health Professionals Follow-up Study (HPFS) showed a significantly positive association between citrus consumption and risk of malignant melanoma (21). Conversely, no evidence of significant association was found in the population from either the Women's Health Initiative (WHI) or National Institutes of Health and the AARP (NIH-AARP) (19, 20). The European Prospective Investigation into Cancer and Nutrition cohort (EPIC) reported that high consumption of citrus fruit, rather than total citrus products, to increase melanoma risk (17). Recently, Marley et al. analyzed data from the UK Biobank cohort, and highlighted the carcinogenic effect of total citrus consumption, especially orange and orange juice intake, on melanoma (18). It is also worth noting that an Italian case-control study suggested high citrus consumption as a protective factor (27).

In general, our findings agree with a carcinogenic role of citrus products in the development of melanoma, support to guide safe sun exposure behaviors among those with high consumption of citrus products. In addition, we indicated the inconsistent influence of citrus consumption on melanoma risk among women and men for the first time. However, the potential mechanisms underlying the sex differences remain to be further explored. More investigation is needed to confirm this finding.

Grapefruit and oranges are two of the most commonly consumed citrus fruits, accounting for more than 90% of citrus market share. Interestingly, grapefruit showed a stronger association with melanoma, which might be explained by its higher content of psoralen when comparing to oranges (28). Industrial processing from fresh fruits to processed juices involves heat, which may decrease the concentration of psoralen (29). Therefore, the null association of the highest consumption of citrus juice with melanoma risk may be a result of processing. However, not all citrus juice loses photocarcinogenicity. Due to its high consumption, orange juice is still significantly associated with melanoma risk (21).

Strengths of our study include comprehensive literature search and large number of participants and cases included. Because we included only prospective cohort studies, the influence of reverse causation and selection bias was minimized. Additionally, either linear or non-linear dose-response analysis between citrus consumption and risk of melanoma was conducted, allowing us to evaluate the shape of the potential association.

Some limitations are worth noting. First, although we identified a few potential sources of heterogeneity, most of the observed high variation among different populations remained unexplained due to limited numbers of studies. Second, the limited original data also resulted in difficulty to the generalizability of our findings. Third, measurement errors in dietary assessment are inevitable and statistical approximation used in out dose-response analyses might also involve errors. Lastly, since our analyses were based on observational studies, residual confounding cannot be completely ruled out.

In conclusion, our dose-response meta-analysis of prospective studies found that higher consumption of total citrus or citrus fruit was associated with an increased risk of melanoma. We also showed an inverted U-shaped relationship between citrus fruit consumption and melanoma risk. These findings have important public health implications with respect to improving melanoma prevention strategies. But extrapolating these conclusions to the global population should be done cautiously, because all results analyzed in the study were reported from Western countries. Thus, further studies are required to strengthen the evidence and to uncover the underlying mechanisms.

## Data Availability Statement

The original contributions presented in the study are included in the article/Supplementary Material, further inquiries can be directed to the corresponding author/s.

## Author Contributions

XF and FL contributed to conception and design. XF and DH searched the databases and checked them according to the eligible criteria and exclusion criteria. JY helped conduct quality assessment of including studies. XF analyzed the data and wrote the draft of the article. Final version was edited and approved by all authors.

## Funding

This study was supported by research grants from the National Natural Science Foundation of China (82170644 and 31900835 to XF; 82074428 to FL), the Natural Science Foundation of Zhejiang Province (Y22C115257 to XF), and the Program of Shanghai Academic Research Leader (20XD1423600 to FL).

## Conflict of Interest

The authors declare that the research was conducted in the absence of any commercial or financial relationships that could be construed as a potential conflict of interest.

## Publisher's Note

All claims expressed in this article are solely those of the authors and do not necessarily represent those of their affiliated organizations, or those of the publisher, the editors and the reviewers. Any product that may be evaluated in this article, or claim that may be made by its manufacturer, is not guaranteed or endorsed by the publisher.
