# Supplementary material for: Citrus Consumption and Risk of Melanoma: A Dose-Response Meta-Analysis of Prospective Cohort Studies
Source: Front Nutr. 2022 Jun 20;9:904957. doi: 10.3389/fnut.2022.904957 (PMC9251443; doi:10.3389/fnut.2022.904957)
Supplement: Supplementary file 1 [file Table_1.docx]

**SUPPLEMENTARY INFORMATION**

**Citrus Consumption and Risk of Melanoma: A Dose-Response Meta-Analysis of Prospective Cohort Studies**

Xuexian Fang, Dan Han, Jun Yang, Fulun Li, Xinbing Sui

**This file includes:**

Supplementary Figure 1

Supplementary Table 1


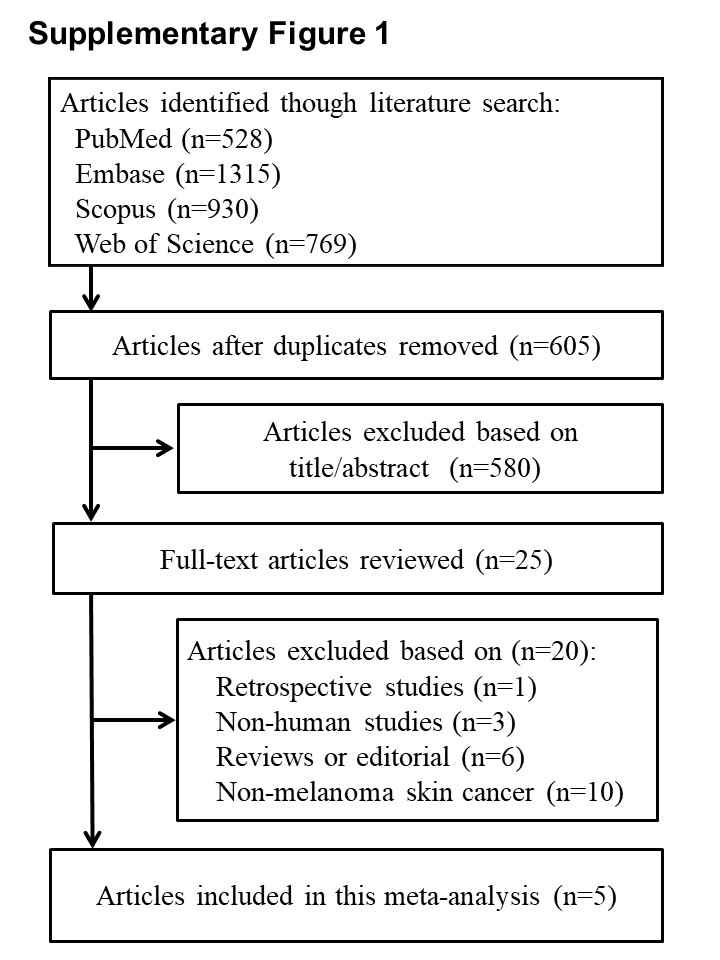


**Supplementary Figure 1.** Flow-chart depicting the literature search and selection strategy.

**Supplementary Table 1.** Quality assessment of all included prospective cohort studies.

| **Author, year** | **Selection** | | | | **Comparability** | **Outcome** | | | **Overall quality** |
| --- | --- | --- | --- | --- | --- | --- | --- | --- | --- |
|  | **Representative of cases** | **Selection of controls** | **Exposure ascertainment** | **No history of disease** | **Comparable on confounders** | **Outcome assessment** | **Adequate follow-up** | **Follow-up rate (> 80%)** |  |
| Mahamat‑Saleh *et al.*, 2020 | 1 | 1 | 1 | 1 | 2 | 1 | 1 | 1 | 9 |
| Marley *et al.*, 2021 | 1 | 1 | 1 | 1 | 2 | 1 | 0 | 1 | 8 |
| Melough *et al.*, 2020 | 1 | 1 | 1 | 1 | 2 | 1 | 1 | 1 | 9 |
| Melough *et al.*, 2021 | 1 | 1 | 1 | 1 | 2 | 1 | 1 | 1 | 9 |
| Wu *et al.*, 2015 | 0 | 1 | 1 | 1 | 2 | 1 | 1 | 1 | 8 |

**Average: 8.6**
